# Epigenetic modifiers to enhance the efficacy of immune checkpoint inhibitors for the treatment of melanoma

**DOI:** 10.1016/j.tranon.2025.102452

**Published:** 2025-06-23

**Authors:** Luigi Liguori, Angelo Luciano, Valentina Pagliara, Giovanna Polcaro, Rosario De Feo, Angela Viggiano, Fabio Salomone, Annarita Avanzo, Filippo Vitale, Simeone D’ambrosio, Alberto Servetto, Cristina R. Ferrone, Stefano Pepe, Francesco Sabbatino

**Affiliations:** a Department of Medicine, Surgery and Dentistry “Scuola Medica Salernitana”, University of Salerno, Baronissi 84031, Italy; b Department of Clinical Medicine and Surgery, University of Naples Federico II, Naples 80131, Italy; c Department of Surgery, Cedars-Sinai Medical Center, Los Angeles 90048, CA, USA

**Keywords:** DNMT, Epigenetic, HDAC, ICI, Melanoma, PD-1, PD-L1, Resistance

## Abstract

Immune checkpoint inhibitor (ICI)-based immunotherapy has significantly improved survival outcomes and radically changed the management of many different types of solid tumors, including melanoma. Almost half of advanced melanoma patients achieve a long-term clinical benefit from ICIs. Unfortunately, the other half of treated patients either rapidly progress or achieve a short-term clinical benefit due to the development of resistance. As a result, novel therapeutic strategies are urgently needed to overcome ICI resistance.

Epigenetic alterations affecting the tumor neoantigen presentation system or modifying the tumor microenvironment can mitigate ICI resistance.

Several studies have demonstrated that epigenetic alterations are potentially targetable by epigenetic modifiers, laying the foundation for the development of novel therapeutic strategies to overcome ICI resistance.

In the present work, we summarize the major epigenetic alterations involved in the pathogenesis of melanoma and their role in ICI resistance. Preclinical evidence supporting the biological rationale for combining epigenetic modifiers with ICIs and an updated overview of the main clinical trials testing epigenetic modifiers for the treatment of melanoma patients will be presented.

## Highlights

• Novel therapeutic strategies are needed to overcome ICI resistance in melanoma.
• Epigenetic alterations play a key role in melanoma pathogenesis and ICI resistance.
• Preclinical data support combining epigenetic modifiers with ICIs.
• Clinical trials may confirm epigenetic modifiers and ICIs as a new melanoma therapy.

## Graphical abstract

**Figure:**
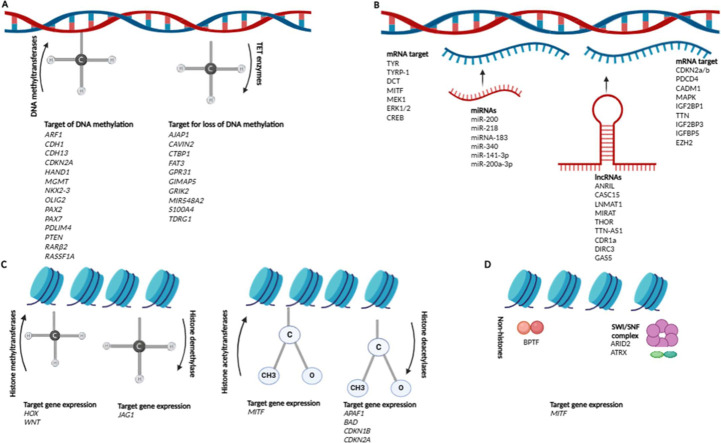

## Introduction

Melanoma is the most aggressive type of skin cancer, accounting for about 100,000 new diagnoses and 8000 deaths per year in the USA [1]. Over the past few years, immune checkpoint inhibitors (ICIs) targeting programmed cell death 1 (PD-1), its ligand 1 (PD-L1) and cytotoxic T-lymphocyte antigen-4 (CTLA-4) have radically changed the management of many types of solid tumors including melanoma [2]. These immune modulating drugs significantly improve the survival outcomes, as well as the quality-of-life of melanoma patients as compared to standard therapies [2]. Consequently, the administration of ICIs as single-agents or in combination with other ICIs represents the standard-of-care for advanced melanoma patients [[3], [4], [5], [6], [7], [8], [9], [10]]. Notably, more than half the patients treated with the combination of ipilimumab (anti-CTLA-4) and nivolumab (anti-PD-1) achieved a long-term clinical benefit. However, the remaining patients either experience rapid disease progression due to innate or primary ICI resistance or patients achieve a short-term clinical benefit due to acquired or secondary ICI resistance [[3], [4], [5], [6], [7], [8], [9], [10], [11], [12],^2^,[11], [12], [13]]. Unfortunately, many mechanisms contribute to primary or secondary ICI resistance including genetic and epigenetic alterations affecting the tumor neoantigen presentation system, or the composition of the tumor microenvironment [14]. As a result, epigenetic modifiers [15,16], compised of a heterogeneous class of drugs that targets the epigenome, exert immunomodulatory effects that may potentially overcome ICI resistance [17,18].

In the present work, we summarize the major epigenetic alterations involved in the pathogenesis of melanoma and their role in ICI resistance. Preclinical evidence supporting the biological rationale for combining epigenetic modifiers with ICIs and an updated overview of the main clinical trials testing epigenetic modifiers for the treatment of melanoma patients will be presented.

## Epigenetic alterations in melanoma pathogenesis

**Fig. 1:**
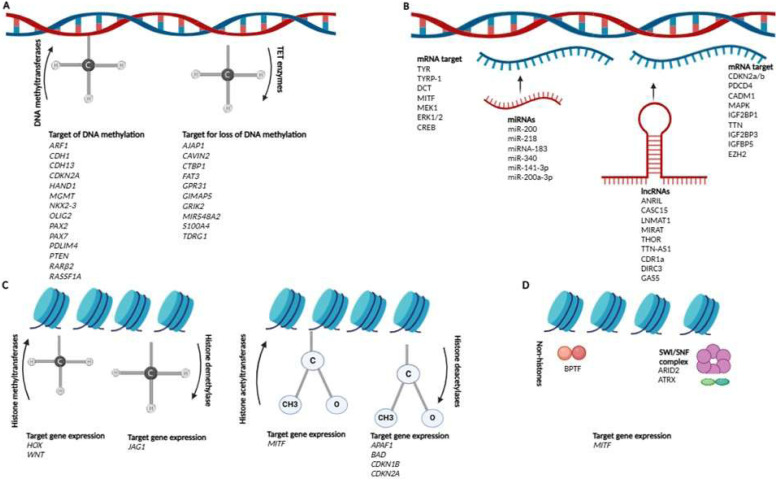
Main mechanisms driving epigenetic alterations involved in the pathogenesis of melanoma include: A) DNA methylation/demethylation; B) Modulation of gene expression by non-coding RNAs (miRNAs and lncRNAs); C) Histone methylation/demethylation and acetylation/deacetylation; and D) Modifications of non-histone proteins.

Epigenetic alterations are defined as reversible and transmissible changes in gene expression without structural modifications in the DNA sequence [19,20]. A growing body of evidence has demonstrated that these alterations are involved in the pathogenesis of melanoma [21,22]. The main mechanisms driving epigenetic alterations in melanoma include: i) DNA methylation/demethylation; ii) modulation of gene expression by non-coding RNAs; iii) histone methylation/demethylation and acetylation/deacetylation; and iv) modifications of non-histone proteins [19,20] (Fig. 1).

### DNA methylation/demethylation

DNA methylation results in the addition of methyl groups at cytosines localized in specific short DNA regions enriched for cytosine-guanine dinucleotides, defined as CpG islands [23]. Almost half of human genes contain CpG islands in their promoter regions and are susceptible to methylation [23,24]. Hypermethylation of CpG islands, mostly mediated by DNA methyltransferases (DNMTs), can silence the expression of tumor suppressor genes, thereby fostering cancer development and progression [19].

Gene methylation significantly contributes to the pathogenesis of melanoma [19]. For instance, gene silencing via hypermethylation of the cyclin-dependent kinase inhibitor 2A gene (*CDKN2A*), a well-known cell- cycle regulator, is described in 19 %-of primary and 33 % of metastatic melanomas [25]. Other tumor suppressor genes frequently hypermethylated in melanoma include phosphatase and TENsin homolog (*PTEN*), ADP Ribosylation Factor 1 (*ARF1*) and RAS association domain family protein 1 (*RASSF1A*) [[26], [27], [28]], Cadherin 1 (*CDH1*), PDZ And LIM Domain 4 (*PDLIM4*), Heart And Neural Crest Derivatives Expressed 1 (*HAND1*), NK2 Homeobox 3 (*NKX2–3*), Oligodendrocyte Transcription Factor 2 (*OLIG2*), O-6-Methylguanine-DNA Methyltransferase (*MGMT*), Cadherin 13 (*CDH13*), Retinoic Acid Receptor Beta (*RARβ2*), Paired Box 2 (*PAX2*) and Paired Box 7 (*PAX7*) [29], as well as other genes. Based on the role of hypermethylation-mediated silencing of tumor suppressor genes in the pathogenesis of melanoma some researchers have suggested that highly hypermethylated tumors be classified as a CpG island methylator phenotype (CIMP) [30]. Currently, the therapeutic algorithm of melanoma patients has not been altered based on their methylation status (CIMP versus No CIMP).

Cancer development and progression is driven by hypermethylation as well as other alterations in oncogenic genes [26,31]. For instance, hypermethylation of *NF1* has been associated with both *NF1* or *RAS* mutations; hypermethylation of *PTEN* with *BRAF* mutations, while no association has been identified between *CDKN2A/B* hypermethylation and alterations of *BRAF, RAS* and *NF1* [32].

Besides hypermethylation of tumor suppressor genes, the pathogenesis of melanoma can also be driven by hypomethylation (mainly mediated by Ten-eleven Translocation (TET) enzymes) of proto-oncogenes as well as of other genes involved in multiple cellular processes such as Adherens Junctions Associated Protein 1 (*AJAP1*), Caveolae Associated Protein 2 (*CAVIN2*), C-Terminal Binding Protein 1 (*CTBP1*), FAT Atypical Cadherin 3 (*FAT3*), G Protein-Coupled Receptor 31 (*GPR31*), GTPase, IMAP Family Member 5 (*GIMAP5*), Glutamate Ionotropic Receptor Kainate Type Subunit 2 (*GRIK2*), S100 Calcium Binding Protein A4 (*S100A4*) and Testis Development Related 1 (*TDRG1*) [33]. As a result, the comprehensive analysis of the whole “methylome” status is essential to decipher the mechanisms underlying melanoma pathogenesis.

### Non-coding RNAs

Non-coding RNAs are untranslated transcripts that modulate gene expression by binding to specific response elements within target mRNA, without altering the DNA sequence [34]. High levels of specific non-coding RNAs can promote cancer development and progression by dysregulating the expression of genes involved in cell cycle regulation [34]. Non-coding RNAs with a length of 20–25 nucleotides are defined as microRNAs (miRNAs) while those exceeding 200 nucleotides are classified as long non-coding RNAs (lncRNAs) [35,36]. Studies have demonstrated that epigenetic alterations mediated by non-coding RNAs may contribute to melanoma pathogenesis [[37], [38], [39]]. Key miRNAs involved in this process include miR-200, miR-218, miR-183, miR-340, miR-141–3p and miR-200a-3p The latter modulate the expression of genes such as *CDH1*, Tyrosinase (*TYR*), Tyrosinase Related Protein 1 (*TYRP-1*), Dopachrome Tautomerase (*DCT*), Melanocyte inducing Transcription Factor (*MITF*), Mitogen-Activated Protein Kinase (*MAPK1/3*), and CAMP Responsive Element Binding Protein 1 (*CREB*) [40,41]. Similarly, specific lncRNAs such as ANRIL, CASC15, LNMAT1, MIRAT, THOR, TTN-AS1, CDR1a, DIRC3 and GAS5 modulate critical melanoma-associated genes, including *CDKN2a/b*, Programmed Cell Death 4 (*PDCD4*), Cell Adhesion Molecule 1 (*CADM1*), *MAPK*, Insulin Like Growth Factor 2 MRNA Binding Protein 1 (*IGF2BP1*) and Titin (*TTN*), Insulin Like Growth Factor 2 MRNA Binding Protein 3 (*IGF2BP3*), Insulin Like Growth Factor 2 MRNA Binding Protein 5 (*IGFBP5*) and Enhancer Of Zeste 2 Polycomb Repressive Complex 2 Subunit (*EZH2*) [42].

### Histone modifications

Histone modifications, mainly methylation/demethylation and acetylation/deacetylation, can influence chromatin accessibility and in turn regulate gene expression [43,44].

Methylation of lysine residues on histone N-terminal tails, mediated by histone methyltransferases (HMTs), can dysregulate the expression of genes involved in melanoma development and progression [21,45]. For instance, *Ceol* et al. demonstrated that SET Domain Bifurcated Histone Lysine Methyltransferase 1 (SETDB1) promotes the development of melanoma by methylating histone H3 at lysine 9 (H3K9), thereby disrupting Homeobox (*HOX*) gene expression [46].

Similarly, *Kato* et al. identified Euchromatic Histone Lysine Methyltransferase 2 (EHMT2) as a promoter of melanoma pathogenesis through the inhibition of DKK1 via H3K9 mono- and di-methylation. The suppression of DKK1 activates WNT signaling, fostering melanoma cell growth. It is noteworthy that this process also promotes melanomas with a “cold” tumor microenvironment (45).

Conversely, the role of histone demethylation in the pathogenesis of melanoma is less well understood. *Roesch* et al. have suggested that the histone demethylase JARID1B sustains tumor growth during the early phase of melanoma development by modulating Notch ligand Jagged 1 (*JAG1*) expression [47]. However, further investigations are needed to elucidate the role of histone demethylation throughout the different phases of melanoma pathogenesis.

In contrast to the limited evidence regarding histone demethylation's impact on melanoma pathogenesis, several studies have demonstrated a role of specific histone acetylation/deacetylations in melanoma pathogenesis. Modulation of histone acetylation is mediated by histone acetyltransferases (HATs) and histone deacetylases (HDACs). Generally, histone acetylation is associated with gene upregulation while histone deacetylation is associated with gene downregulation [48]. *Fiziev* et al. reported that loss of histone acetylation at regulatory regions of key tumor suppressor genes, including BCL2 associated agonist of cell death (*BAD*), CDKN2A, Cyclin-dependent kinase inhibitor 1B (*CDKN1B*) and apoptotic protease activating factor-1 (*APAF1*), is pivotal in melanoma development. Notably, restoring acetylation levels at deacetylated loci using HDAC inhibitors (HDACis) suppressed aberrant melanoma cell proliferation [49]. Conversely, CBP/p300-mediated acetylation of histone H3 enhances melanoma cell growth by promoting the transcription of *MITF*, a key regulator of melanocyte survival [48]. As a result, an "imbalanced acetylation status," rather than isolated acetylation or deacetylation events, significantly impact the pathogenesis of melanoma [45,[49], [50], [51]].

### Non-histone protein modifications

Non-histone proteins, that compose the chromatin remodeling complexes, are needed for the modulation of gene expression by promoting dynamic access to packaged DNA and tailoring nucleosome composition [52]. Some lines of evidence demonstrate that different levels of expression or mutations in these non-histone proteins can influence melanoma pathogenesis [53]. For instance, the overexpression of Bromodomain PHD Finger Transcription Factor (BPTF), a subunit of the NURF chromatin remodeling complex, is reported to foster the proliferation of melanoma cells, [54,55] as well as predict a poor prognosis in melanoma patients [55]. In contrast, the silencing of BPTF reduces the proliferative capacity and metastatic potential of melanoma cells [55]. *Koludrovic* et al. have demonstrated that BPTF modulates this process by promoting the overexpression of *MITF* [54]. The latter plays a key role in melanocyte survival and progression by modulating the expression of different proteins required for melanin synthesis, including TYR, TYRP1 and DCT [[55], [56], [57], [58], [59]].

Similarly, mutations in the components of the SWI/SNF chromatin remodeling complex, such as AT-rich interactive domain-containing protein 2 (ARID2) and ATRX Chromatin Remodeler (ATRX) have been identified in melanoma samples [60,61]. However, how mutations in components of SWI/SNF promote the pathogenesis of melanoma remains to be defined.

Despite significant advances in understanding the role of epigenetic alterations, the interplay between diverse epigenetic alterations throughout the multistep process of melanoma pathogenesis is not fully understood. Further studies are needed to elucidate their collective impact on the development and progression of melanoma.

## Epigenetic alterations as mechanisms of resistance to ICI-based immunotherapy

Tumor-intrinsic ICI resistance mechanisms can include alterations in the DNA damage response, activation of oncogenic signaling pathways, and/or promotion of immune evasion techniques, while tumor-extrinsic mechanisms encompass immunosuppressive modifications of the tumor microenvironment [[62], [63], [64]]. Recent preclinical studies have suggested that both tumor-intrinsic and -extrinsic mechanisms of ICI resistance can be epigenetically mediated [22,65].

In this context, the imbalances in DNA or histone methylation status have been reported in various models of ICI resistance. For instance, *Sheng* et al. demonstrated that dysfunction of histone lysine-specific demethylase 1 (LSD1) in cancer cells impacts anti-tumor T cell immunity by modifying the intra-tumoral CD8+ *T* cell infiltration [66]. Indeed, the levels of LSD1 expression in cancer cells are inversely correlated with intra-tumoral CD8+ *T* cell infiltration, a well-known tumor-extrinsic mechanism of ICI resistance [67]. As result, overexpression of LSD1 may promote ICI resistance [68].

Similarly, dysregulation of histone methyltransferase EZH2 has been involved in tumor-extrinsic resistance [69,70]. Indeed, *Zingg* et al. reported that overexpression of histone methyltransferase EZH2 in melanoma cells contributes to ICI resistance by decreasing the intra-tumoral interferon-γ-producing PD-1^low^ CD8^+^ *T* cell infiltration. Additionally, overexpression of histone methyltransferase EZH2 can contribute to tumor-intrinsic ICI resistance by impairing antigen presentation, as well as by promoting the upregulation of PD-L1 expression on melanoma cells [70]. These findings highlight the potential of targeting LSD1 or EZH2 to overcome ICI resistance.

The upregulation of PD-L1 expression in melanoma cells is a well-known tumor-intrinsic mechanism [71]. This process can also be epigenetically mediated [72,73]. *Lienlaf* et al. demonstrated that histone deacetylase 6 (HDAC6) modulates the expression of PD-L1 by activating the STAT3 pathway [74]. Specifically, the overexpression of HDAC6 increases the activity of STAT3-mediated signaling, which in turn fosters the upregulation of PD-L1 expression on melanoma cells. In contrast, the inhibition of HDAC6 decreases the expression of many immune checkpoint molecules such as PD-L1. This data suggests that the inhibition of HDAC6 may represent a novel potential strategy to overcome ICI resistance.

Despite this solid rationale and preclinical data, few clinical studies have been performed to validate its potential clinical efficacy. *Seremet* et al. demonstrated that the methylation profile can influence the tumor response of melanoma patients treated with ipilimumab-based immunotherapy. They demonstrated that two specific methylation profiles in genes regulating the development and differentiation of the nervous system are correlated with tumor response, as well as with specific tumor characteristics [75]. The different methylation profiles were associated with CD8+ *T* and PD-L1+ cell infiltration, as well as with focal loss of HLA class I and TAP-1 expression. The latter were significantly correlated with patient response to ICIs. However the impact of the methylation status on patient response to ICI and therefore on ICI resistance needs to be further validated [75].

## Preclinical evidences of the combination of epigenetic modifiers and ICI-based immunotherapy

In view of the role of epigenetic changes in ICI resistance, some researchers have suggested that targeting these epigenetic alterations with epigenetic modifiers could enhance the efficacy of ICI-based immunotherapy.

Although, no selective drugs targeting specific epigenetic alterations are currently available, some non-selective epigenetic modifiers have demonstrated “pro-immunomodulatory” effects to enhance the anti-tumor immune response [76]. Some of these effects target tumor-intrinsic mechanisms of ICI resistance while others target tumor-extrinsic mechanisms.

For instance, *Woods* et al., demonstrated that HDACis induce robust and durable upregulation of PD-L1 expression on tumor cells both in vitro and in a syngeneic murine model [77]. As confirmed by chromatin immunoprecipitation (ChIP) analyses, this effect is directly mediated by the increased levels of histone H3 acetylation in the promoter region of *PD-L1*. The dynamic induction of PD-L1 expression enhances ICI efficacy more effectively than basal PD-L1 expression [78]. Due to these results, the same authors revealed that combining HDACis with anti-PD-1 inhibitors significantly slows melanoma progression and increases survival as compared to single-agent treatments in vivo [77].

Alterations in tumor antigen presentation represents an important tumor-intrinsic mechanism of ICI resistance. Cancer cells evade the anti-tumor immune response by decreasing their tumor associated antigen (TAAs) expression. Recent studies demonstrated that DNMTis and HDACis can restore cancer cell recognition by T cells by increasing the expression of TAAs, such as cancer testis antigens (CTAs) [76,79]. The latter are typically expressed in germ cells while silenced by methylation in somatic or cancer cells [80]. The overexpression of CTAs or other TAAs may in turn promote cancer cell recognition by T cells [81]. As a result, the removal of methylation-mediated silencing, DNMTis, promotes the overexpression of CTAs/TAAs, including MAGE-A1 and NY-ESO-1 which enhances T cell-mediated killing, and provides a rationale for combining DNMTi with ICIs [[82], [83], [84], [85]]. Similar effects have been observed with HDACis [86,87]. For instance, by removing acetylation-mediated silencing, romidespin enhances the expression of the gp100 protein in human melanoma cells promoting T cell-mediated killing [86].

In addition, DNMTis such as azacytidine and decitabine can also promote an anti-tumor immune response by upregulating the antigen processing machinery (APM) HLA class I components such as β2-microglobulin, HLA-A-B-C antigens, PSMB8, PSMB9 and TAP1 [84,85,88]. Similar results were obtained with HDAC6is, such as tubastatin A and nexturastat A, which upregulate both HLA class I and the expression of specific melanoma associated antigens (gp100, MART1, TYRP1 and TYRP2) both in human and murine melanoma cells [87]. These findings suggest that both DNMTis and HDACis promote the mounting of an effective ICI-mediated anti-tumor response by improving tumor antigen presentation.

On the other hand, epigenetic modifiers can also target tumor-extrinsic mechanisms of ICI resistance. For instance, *Sheng* et al. demonstrated that the overexpression of the histone demethylase LSD1 decreases intra-tumoral CD8+ *T* cell infiltration and increases their exhaustion. Noteworthy, inhibiting LSD1 with small molecules increases the infiltration of intra-tumoral CD8+ *T* cells and prevents their exhaustion by inducing the production of endogenous retroviral elements (ERVs), leading to double-stranded RNA (dsRNA) stress and activation of the type I interferon pathway. The latter mediates many aspects of the immune anti-tumor response including intra-tumoral CD8+ *T* cell infiltration [66]. Upregulating the type I interferon pathway has been demonstrated to be effective in enhancing the efficacy of ICI-based immunotherapy [89]. This finding, was confirmed by in vivo experiments, validated by the sequential use of LSD1 inhibitors and anti-PD-1 inhibitors as a potential strategy for effective and more durable responses to anti-PD-1 inhibitors in melanoma [66,90].

In the context of the anti-tumor immune response unleashed by anti-PD-1/PD-L1-based immunotherapy, interferon-gamma (IFN-γ) plays a major role. Dysregulation of IFN-γ is a key mechanism of tumor-extrinsic resistance, often driven by hypermethylation [91]. Emerging evidence demonstrated that DNMTis and EZH2is can restore the IFN-γ pathway by reversing aberrant methylation patterns [92,93]. *Tiffen* et al. have reported that histone methylase EZH2, together with DNA methyltransferase, cooperates to downregulate both IFN-γ and IFN-α pathways resulting in ICI resistance. They also demonstrated that EZH2is and DNMTis can reverse this phenomenon restoring an anti-cancer immune response which is mediated by interferon pathways [92]. The molecular signal transduction mechanisms underlying this finding were investigated using genome-wide DNA methylation profiling. *Falahat* et al. [94] demonstrated that hypermethylation of the promoter of the stimulator of interferon gene (*STING*) resulted in downregulation of the IFN-mediated anti-cancer immune response in melanoma cell lines. Moreover, in co-colture models of HLA-matched tumor infiltrating lymphocytes (TILs) and melanoma cells, DNMTis which restore the STING pathway, promote the upregulation of HLA class I components which are essential for the recognition of cancer cells by T cells. As a result, these findings suggest that therapeutic strategies including DNMTis to enhance antigen presentation through STING pathway upregulation may improve the efficacy of ICI-based immunotherapy [95].

Lastly, epigenetic modifiers can reshape the characteristics of the tumor microenvironment by modulating the development and differentiation of specific pro-immunogenic immune cells [[96], [97], [98], [99]]. Specifically, *Wang* et al. demonstrated that low dose decitabine demethylates the promoter region of *CD8 and,* enhances the infiltration and the cytotoxic effect of CD8+ *T* cells [100]. decitabine can also induce a robust proliferation and activation of NK cells by increasing the expression of the Killer Ig-like receptor (KIR) and the NKp44 receptor [101]. Many studies demonstrated that tumors characterized by increased intra-tumoral infiltration of activated CD8+ and NK cells (termed as “inflamed” tumor microenvironment), are more susceptible to ICI-based immunotherapy [102,103]. Therefore, decitabine’s ability to modulate the tumor microenvironment composition represents a potential option for enhancing ICI treatment in melanoma.

Similarly, HDACis promote an increased CD8+ *T* cell infiltration as well as M1 macrophage polarization within the tumor microenvironment. These effects, mediated by the degradation of acetylated *c-Myc*, are well-known to shift the tumor microenvironment from an immunosuppressive to a pro-immunogenic state [104,105]. Murine models have further confirmed that this reprogramming mediates the synergistic effect between HDACis and anti-PD-1-based immunotherapy [106]. Conversly, the presence of immunosuppressive cells such as regulatory T (Treg) FOXP3+ cells can impair the anti-tumor response which is correlated with a poor prognosis in melanoma patients treated with ICIs [[107], [108], [109]]. Both in vitro and *vivo* experiments demonstrated that EZH2 plays a significant role in Treg cell regulation. Indeed, the inhibition of EZH2 decreases FOXP3 expression on Treg cells, mitigating their immunosuppressive activity and promoting the recruitment of CD8+ *T* cells within the tumor microenvironment. As a result, EZH2 inhibitors represent a potential breakthrough strategy to enhance the efficacy of ICI-based immunotherapy by reshaping the characteristics of the tumor microenvironment towards a pro-immunogenic phenotype, [110].

Collectively, these findings highlight the complex interplay between epigenetic modifications and immune signaling pathways in melanoma landscape. By using epigenetic modifiers, it is possible to modulate immune checkpoint expression, enhance tumor antigen presentation, and reshape the tumor microenvironment composition to promote immune anti-tumor activity. These insights provide a compelling biological rationale for epigenetic modifiers to enhance the efficacy of ICIs.

## Epigenetic modifiers for the treatment of melanoma patients

Recent advances in understanding the role of epigenetic alterations in melanoma pathogenesis and ICI resistance have paved the way for exploring epigenetic modifiers as therapeutic agents in melanoma patients as a single agent or in combination with chemotherapy, targeted therapy, or ICIs [111]. These drugs aim to reverse epigenetic alterations in order to restore normal gene expression patterns and enhance anti-tumor immune responses [111]. The main types of epigenetic modifiers tested in melanoma clinical trials include: i) DNMT inhibitors (DNMTis); ii) HDACis; and iii) Enhancer Of Zeste 2 Polycomb Repressive Complex 2 Subunit inhibitor (EZH2is).

### Clinical evidence of epigenetic modifiers as a single agent or in combination with chemotherapy or targeted therapy

Among DNMTis, azacitidine and decitabine (currently approved for the treatment of myelodysplastic syndrome [[112], [113]]) have been tested in melanoma clinical trials. However, scant results have been achieved or reported (Table 1).

**Table 1:** Clinical trials testing the utilize of epigenetic modifiers as single-agent therapy or in combination with other agents for the treatment of melanoma patients.

Agent	Phase	Condition	Outcomes	Status	ClinicalTrials.gov ID
**DNA methyltransferase inhibitors (DNMTis)**					
decitabine (Dacogen)	I	Unresectable stage III or IV melanoma	NA	Completed	NCT00002980
decitabine (Dacogen) in combination with temozolomide	I/II	Metastatic melanoma	ORR: 18 % DCR: 61 % mPFS: 3.4 months mOS: 12.4 months	Completed	NCT00715793
decitabine (Dacogen) in combination with vemurafenib and cobimetinib	I	Previously treated BRAF ^V600E^ mutated advanced melanoma	NA	Terminated	NCT01876641
decitabine (Dacogen) in combination with peg-interferon alfa-2b	I/II	Unresectable stage III or IV melanoma	NA	Terminated	NCT00791271
Azacitidine in combination with interferon alfa-2b	I	Metastatic melanoma	NA	Completed	NCT00398450
Azacitidine in combination with interferon alfa-2b	I	Unresectable stage III or IV melanoma or stage IV kidney cancer	NA	Completed	NCT00217542
**Histone deacetylase inhibitors (HDACis)**					
Entinostat	II	Unresectable stage III or IV non-uveal melanoma	ORR: 0 %	Completed	NCT00185302
Depsipeptide	II	Unresectable stage III or IV melanoma	NA	Terminated	NCT00104884
Panobinostat	I	Unresectable stage III or IV	ORR: 0 % DCR: 27 %	Completed	NCT01065467
Panobinostat in combination with decitabine (Dacogen) and temozolomide	Ib/II	Previously treated metastatic melanoma	DCR: 75 %	Completed	NCT00925132
valproic acid (Depakene) in combination with karenitecin (Topoisomerase I inhibitor)	I/II	Metastatic melanoma	NA	Terminated	NCT00358319
valproic acid (Depakene) in combination with neratinib (HER1–2–4 inhibitor)	I/II	Advanced solid tumors including RAS-mutated tumors, EGFR-altered GBM and ocular melanoma	NA	Recruiting	NCT03919292
Vorinostat	I/II	Previously treated BRAF ^V600E^ mutated advanced melanoma	NA	Unknown	NCT02836548
Vorinostat	II	Unresectable stage III or IV melanoma	mPFS: 5 months	Completed	NCT00121225
Vorinostat	II	Metastatic uveal melanoma	NA	Active, not recruiting	NCT01587352
Vorinostat in combination with marizomib (proteasome inhibitor)	I	Metastatic NSCLC, pancreatic cancer, melanoma or lymphoma	NA	Completed	NCT00667082
**Enhancer of zeste 2 polycomb repressive complex 2 subunit inhibitor (EZH2i)**					
Tazemetostat in combination with dabrafenib and trametinib	I/II	Previously treated BRAF ^V600^ mutated metastatic melanoma	NA	Recruiting	NCT04557956

List of abbreviations: BRAF: V-raf murine sarcoma viral oncogene homolog B, DCR: sisease control rate, DNMTi: DNA methyltransferase inhibitor, EGFR: Epidermal growth factor receptor, EZH2i: Enhancer of zeste 2 polycomb repressive complex 2 subunit inhibitor, GBM: Glioblastoma multiforme, HDACi: Histone deacetylase inhibitor, HER: human epidermal growth factor receptor, mOS: median overall survival, mPFS: median progression-free survival, NA: Not available, NSCLC: Non-small cell lung cancer, ORR: Objective response rate, RAS: Rat Sarcoma Viral Oncogene Homolog,.

decitabine , both as a monotherapy and in combination with other agents, has been utilized in Phase I and II clinical trials. The phase I basket trial NCT00002980, tested single agent decitabine in previously treated advanced cancer patients, but results have yet to be reported. In contrast, promising results have emerged from clinical trials exploring decitabine -based combinatorial strategies. In the phase I/II NCT00715793 clinical trial, the combination of decitabine and temozolomide was tested in previously treated patients with metastatic melanoma. The objective response rate (ORR), disease control rate (DCR), median progression-free survival (mPFS) and median overall survival (mOS) were 18 %, 61 %, 3.4 months and 12.4 months, respectively. All grade and serious adverse event rates were 100 % and 17.95 %, respectively.

The phase I NCT01876641 clinical trial BRAF inhibitor-resistant melanoma patients were treated with the combination of decitabine and vemurafenib (BRAF inhibitor) plus cobimetinib (MEK inhibitor). Preliminary results demonstrated a 43 % of response rate including three patients (21 %) achieving a complete response (CR) [114]. The most common adverse events included hyperbilirubinemia, hypophosphatemia, leukopenia, arthralgia, and rash, most of which were grade 1 or 2 [114]. Unfortunately, this trial was prematurely terminated due to funding issues, and no further studies have been conducted to validate the role of decitabine in combination with BRAF and MEK inhibitors.

No results from the two clinical trials (NCT00398450 and NCT00217542) for the use of Azacitidine in melanoma patients have been reported.

In contrast to DNMTis, more clinical data are available regarding the role of HDACis for the treatment of melanoma patients. These drugs restore the acetylation status and exert additional anti-tumor effects including free radical generation and promotion of apoptosis [115].

HDACis tested in melanoma patients include entinostat, panobinostat and vorinostat. Unfortunately, both entinostat and panobinostat, when used as monotherapy, have shown limited efficacy. In the phase II NCT00185302 clinical trial, patients with unresectable stage III or IV melanoma treated with entinostat, achieved an ORR of 0 % [116]. Similarly, in the phase I NCT01065467 clinical trial, the use of oral panobinostat provided an ORR and a DCR of 0 % and 27 %, respectively. Additionaly, a high rate of adverse events was reported [117]. In contrast, promising results were achieved in the phase Ib/II NCT00925132 clinical trial utilizing panobinostat in combination with both decitabine and temozolomide. A DCR of 75 % and a favourable toxicity profile was reported. However no further validation has been pursued [118].

Promising results have also been reported with vorinostat in the phase II NCT00121225 clinical trial. A median PFS of 5 months (range, 2–8 months) was reported in patients with advanced melanoma. Validation of the obtained results is currently being investigated in the phase II NCT01587352 clinical trial. Vorinostat has also been tested in combination with marizomib (proteasome inhibitor) in the phase I NCT00667082 clinical trial, but no results have been reported, and development of combinatorial strategies involving vorinostat seem to have stalled.

Evidence suggests that valproic acid can reverse the activity of HDACs [119]. As a result, valproic acid was tested as a co-adjuvant for the treatment of melanoma patients. However, the results from the phase I/II NCT00358319 clinical trial, in which patients with metastatic melanoma received a combination of valproic acid with karenitecin (a topoisomerase I inhibitor), are not available due to an early termination of the trial.

Lastly, an ongoing clinical trial is evaluating the combination of the EZH2i tazemetostat with dabrafenib (BRAF inhibitor) and trametinib (MEK inhibitor) in patients with advanced BRAF^V600^ mutant melanoma (NCT04557956). The results of this trial are still pending.

### Combining epigenetic modifiers with ICI-based immunotherapy: update of the clinical evidence

DNMTis and HDACis are currently being combined with ICIs in order to enhance their clinical efficacy and overcome ICI resistance (Table 2).

**Table 2:** Clinical trials testing the combination of epigenetic modifiers and ICI for the treatment of melanoma patients.

Agent	Phase	Condition	Outcomes	Status	ClinicalTrials.gov ID
**DNA methyltransferase inhibitors (DNMTis)**					
decitabine (Dacogen)/​cedazuridine in combination with nivolumab	Ib/II	Metastatic mucosal melanoma	NA	Recruiting	NCT05089370
Azacitidine in combination with pembrolizumab	II	Metastatic melanoma	NA	Active, not recruiting	NCT02816021
Guadecitabine (Dacogen) in combination with ipilimumab	Ib	Unresectable stage III or IV melanoma	mPFS: 5.2 months mOS: 25.6 months	Unknown status	NCT02608437
Guadecitabine (Dacogen) or ASTX 727 (decitabine (Dacogen) plus cedaruzidine) in combination with ipilimumab and nivolumab	II	Metastatic melanoma (cohort A) and NSCLC (cohort B)	Cohort A: ORR: 39 % in combination group vs 17 % ipilimumab-nivolumab group Cohort B: NA	Not yet recruiting	NCT04250246
**Histone deacetylase inhibitors (HDACis)**					
Domatinostat in combination with pembrolizumab	Ib/II	Unresectable stage III or IV melanoma	NA	Completed	NCT03278665
Entinostat in combination with pembrolizumab	II	'Non-inflamed' unresectable stage III or IV melanoma	ORR: 27.3 %	Completed	NCT03765229
Entinostat in combination with pembrolizumab	II	Metastatic uveal melanoma	ORR: 14 % mPFS: 2.1 months mOS: 13.4 months	Completed	NCT02697630
Entinostat in combination with pembrolizumab	I/II	Advanced cancer including CRC, melanoma and NSCLC	NA for melanoma patients	Completed	NCT02437136
Mocetinostat in combination with ipilimumab and nivolumab	Ib	Unresectable stage III or IV melanoma	ORR: 80 % grade 3–4 AE rate: 60 %	Terminated	NCT03565406
Mocetinostat in combination with durvalumab	I/II	Metastatic solid tumors including melanoma and NSCLC	NA for melanoma patients	Terminated	NCT02805660
Panobinostat in combination with ipilimumab	I	Unresectable stage III or IV melanoma	Cohort 5mg Panobinostat:ORR: 16.7 % -mPFS: 4.19 months -mOS: 25.91 months Cohort 10mg Panobinostat:ORR: 9.1 % -mPFS: 1.81 months -mOS: 12 months	Completed	NCT02032810
Panobinostat in combination with spartalizumab	Ib	Metastatic melanoma and NSCLC	NA	Withdrawn	NCT03982134
Tinostamustine in combination with nivolumab	I	Refractory, locally advanced or metastatic melanoma	NA	Unknown status	NCT03903458

List of abbreviations: AE: Adverse event, CRC: Colorectal cancer, DNMTi: DNA methyltransferase inhibitor, HDACi: Histone deacetylase inhibitor, mOS: median overall survival, mPFS: median progression-free survival, NA: Not available, NSCLC: Non-small cell lung cancer, ORR: Objective response rate.

The trials evaluating the efficacy of DNMTis combined with ICIs for the treatment of melanoma patients are still ongoing and few results are currently available.

For instance, the phase II NCT02816021 clinical trial is currently exploring the efficacy of azacitidine and pembrolizumab (anti-PD-1) for advanced melanoma patients; the phase Ib/II NCT05089370 clinical trial, the efficacy of decitabine/cedazuridine and nivolumab (anti-PD1) for metastatic mucosal melanoma.

Long-term results are only available from the phase Ib dose-escalation NCT02608437 clinical trial, where patients with metastatic melanoma received the combination of guadecitabine (30, 45 or 60 mg/m^2^/day subcutaneously on days 1 to 5 every three weeks) and ipilimumab (3 mg/kg intravenously every three weeks). After a 45-month follow-up, median PFS and median OS were 5.2 months and 25.6 months, respectively. The 5-year OS was 28.9 % and the median duration of response was 20.6 months [120]. Translational investigation demonstrated that a specific guadecitabine-signature is correlated with treatment response [120]. The efficacy of this strategy has been recently confirmed by the preliminary results of the NCT04250246 trial. In this phase II clinical trial, anti-PD-1/PD-L1 resistant advanced melanoma patients (cohort A) and non-small cell lung cancer (NSCLC) patients (cohort B) were randomized to receive the combination of ipilimumab and nivolumab with (arm A) or without (arm B) ASTX 727 (an oral combination of decitabine and cedaruzidine). Preliminary results from cohort A showed an ORR of 39 % in arm A compared to 17 % in arm B, with a DCR of 56 % in arm A compared to 33 % in arm B [121]. Any grade adverse events occurred in 94 % of patients in arm A and in 100 % of patients in arm B with grade 3–4 adverse events in 72 % and 50 % of the patients in arm A and arm B, respectively [121]. These promising results need to be confirmed in a Phase III trial with a long-term survival analysis.

On the other hand, more results from clinical trials testing HDACis are available. For instance, entinostat was tested in combination with pembrolizumab in 'non-inflamed' unresectable stage III or IV melanoma patients (NCT03765229 trial). An encouraging ORR of 27.3 % was reported, although the results were limited by the small sample size of the study population. The rate of severe adverse events was 45.45 % [122]. The combination of entinostat and pembrolizumab was also investigated in the phase I/II NCT02437136 clinical trial, however the results from the cohort of melanoma patients are still pending.

Interesting results emerged from the phase Ib NCT03565406 clinical trial, in which patients with unresectable stage III/IV melanoma patients treated with the combination of mocetinostat, ipilimumab and nivolumab, achieved an ORR of 80 %. However, all patients experienced grade 2 or higher adverse events with a grade 3–4 adverse event rate of 60 % [123]. Unfortunately, the sponsor de-prioritized the development of this therapeutic strategy and no further study has been conducted, so far.

Similarly, ipilimumab and spartalizumab (anti-PD-1) were tested in combination with the HDACi panobinostat. In the phase I NCT02032810 clinical trial, two doses of panobinostat (5 mg and 10 mg) were tested in combination with ipilimumab (3 mg/kg) for the treatment of unresectable stage III or IV melanoma patients. ORR, mPFS and mOS were 16.7 %, 4.19 months, 25.91 months and 9.1 %, 1.81 months, and 12.0 months, respectively, in the cohort at 5 and 10 mg. Similar rates of severe adverse events in both groups were reported (66.67 % and 63.64 %). The discrepancy in terms of efficacy between the two cohorts still remains to be clarified as the combination of panobinostat at the most effective dose of 5 mg did not increase the response rate of ipilimumab in advanced melanoma patients [124]. One would hypothesize that different doses of panobinostat may elicit "pro-immunogenic" or immunosuppressive effects to ICIs [125,126]. As a result, further studies are needed.

On the other hand, no results from the phase Ib NCT03982134 clinical trial for the combination of spartalizumab and panobinostat have been reported and the trial was closed.

## Conclusion

Growing evidence supports the critical role of epigenetic alterations in the pathogenesis of melanoma, as well as ICI resistance. Preclinical evidence demonstrates that targeting these alterations is a promising strategy to enhance the efficacy of ICI-based immunotherapy. While the use of epigenetic modifiers as single-agent therapy has not provided significant results for melanoma patients, preliminary results from clinical studies exploring the combination of epigenetic modifiers and ICIs appears to be scant, so far and many challenges remain including i) differences in clinical outcomes between different dosing regimens and types of immunotherapeutic backbones (anti-PD-1 vs anti-CTLA-4 vs anti-PD-1 plus anti-CTLA-4), ii) high rates of grade 3–4 adverse events; and iii) the long-term clinical benefits. The strong rationale for combining epigenetic modifiers with ICIs generates momentum to further test this novel strategy in clinical trials for patients with advanced melanoma.

## CRediT authorship contribution statement

**Luigi Liguori:** Writing – review & editing, Writing – original draft, Methodology, Data curation, Conceptualization. **Angelo Luciano:** Writing – original draft, Data curation. **Valentina Pagliara:** Writing – review & editing. **Giovanna Polcaro:** Writing – review & editing. **Rosario De Feo:** Data curation. **Angela Viggiano:** Data curation. **Fabio Salomone:** Methodology. **Annarita Avanzo:** Data curation. **Filippo Vitale:** Methodology. **Simeone D’ambrosio:** Writing – original draft. **Alberto Servetto:** Writing – review & editing. **Cristina R. Ferrone:** Writing – review & editing. **Stefano Pepe:** Supervision, Funding acquisition. **Francesco Sabbatino:** Writing – review & editing, Writing – original draft, Methodology, Data curation, Conceptualization.

## Declaration of competing interest

All the authors have no competing interests to declare in relation to this paper.
